# Transplantation of Horseshoe Kidney from Living, Genetically Unrelated Donor

**DOI:** 10.1155/2015/390381

**Published:** 2015-07-09

**Authors:** Kazuro Kikkawa, Takahito Wakamiya, Hiroki Kusumoto, Nagahide Matsumura, Yasuo Kohjimoto, Isao Hara

**Affiliations:** Department of Urology, Wakayama Medical University, 811-1 Kimiidera, Wakayama 641-0012, Japan

## Abstract

We report a case of renal transplantation using a horseshoe kidney from a living, genetically unrelated donor. The recipient was a 60-year-old man with diabetic nephropathy, and the donor was the 63-year-old wife of the recipient with a horseshoe kidney free of complications. Computed tomography showed two renal arteries and one renal vein on the left side, and the isthmus was perfused by several accessory arteries and veins. To demarcate the boundary of the isthmus, the left renal artery was ligated and cannulated for in situ perfusion. Furthermore, the isthmus was clamped, and the boundary of the isthmus was confirmed. The kidney was divided at the left margin of the perfused boundary. The cut ends of the isthmus were closed by sutures. The left kidney was transplanted into the right iliac fossa of the recipient. Asymptomatic fluid collection occurred on the cut surface at the isthmus of the donor, and this fluid decreased in due course. On the other hand, the recipient experienced no surgical complication or rejection, while maintaining serum creatinine levels of 2.00–2.20 mg/dL over a 22-month follow-up period. Horseshoe kidneys may be used for transplantation in selected cases after a detailed preoperative evaluation.

## 1. Introduction

The number of patients with chronic kidney disease who are awaiting a transplant is increasing. The continuing donor organ shortage has led to an expansion of the criteria for acceptability of deceased-donor organs, including the use of marginal kidneys with congenital abnormalities.

The horseshoe kidney is one of the most common congenital anomalies. The incidence has been reported to range from 1 per 600–800 [1]. The vascular anatomy in the horseshoe kidney is complex, and ureteral abnormalities also may be present. Therefore, urinary tract infection, vesicoureteral reflux, nephrolithiasis, and hydronephrosis are frequent complications in patients with horseshoe kidneys.

Although several deceased-donor kidney transplantations have been performed to date, use of divided horseshoe kidneys from living donors is rare. We present our experience with a horseshoe kidney used as a renal transplant from a living, genetically unrelated donor.

## 2. Case Report

**Figure 1:**
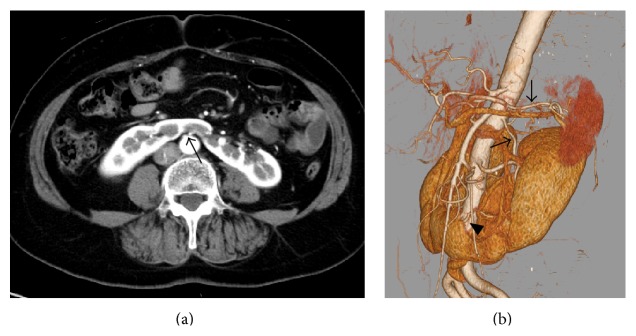
(a) Preoperative donor CT images with contrast enhancement showed the isthmus of the horseshoe kidney and an artery from the aorta supplying the isthmus (arrow). (b) Three-dimensional reconstruction of the CT scan of the donor revealed various blood vessels supplying the horseshoe kidney. Two left renal arteries (arrows) supplied the upper and middle poles of the left kidney and two aortic branches (arrowheads) supplied the isthmus.

**Figure 2:**
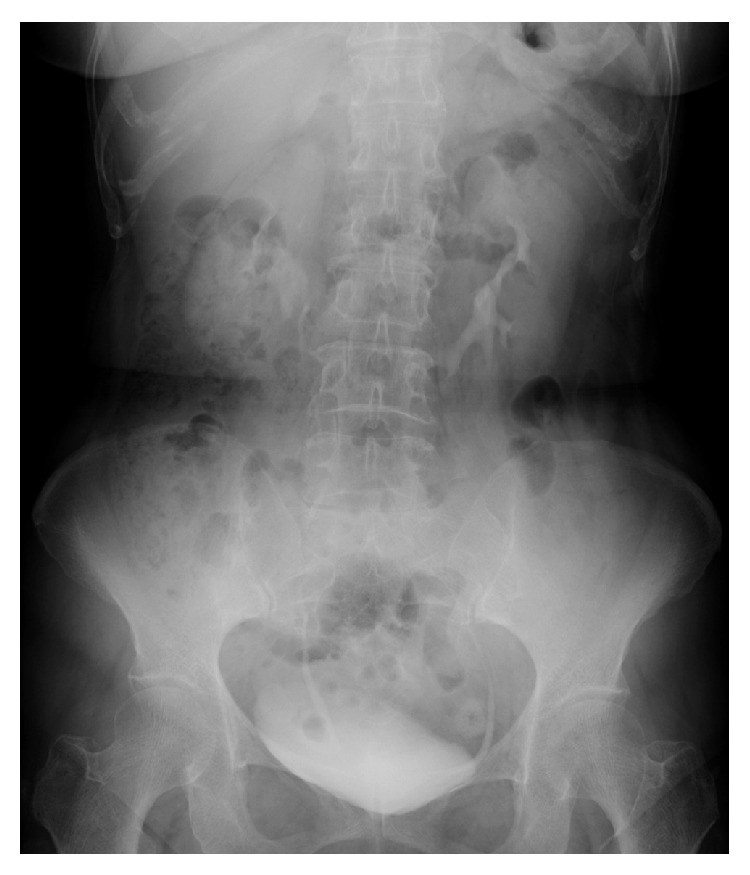
Intravenous urography of the donor revealed a medially rotated pelvis. Bilateral collecting systems were not connecting, and hydronephrosis was not seen.

The recipient was a 60-year-old man with end-stage renal disease related to diabetic nephropathy. The donor was the 63-year-old wife of the recipient. Preoperative computed tomography (CT) revealed a horseshoe kidney with no hydronephrosis or calculi in the donor (Figure 1(a)). Dynamic CT showed one renal artery and one renal vein on the right side, two renal arteries and one renal vein on the left side, and several accessory arteries and veins distributed to the lower fused parenchyma (Figure 1(b)). Intravenous urography showed no communicating calyceal system (Figure 2). Because the renal function of the left kidney was slightly lower than that of the right kidney according to renal scintigraphy, the left kidney was chosen for use as a transplant.

**Figure 3:**
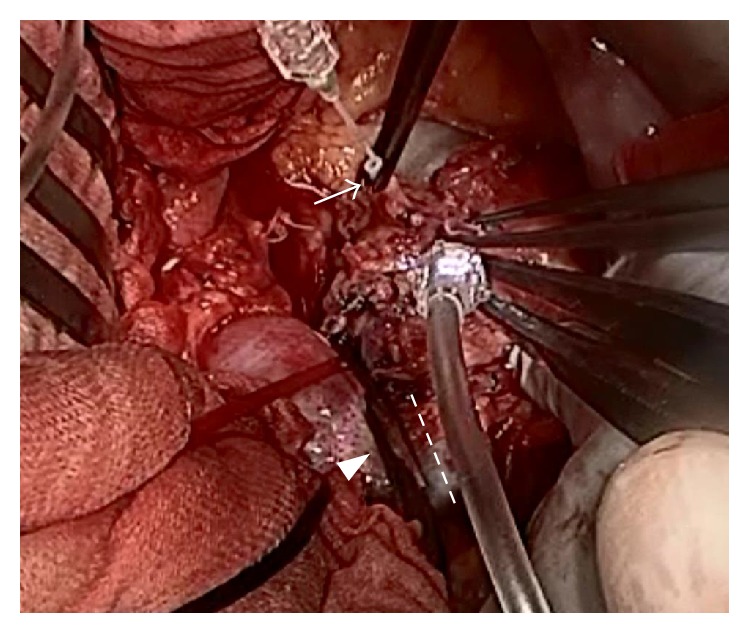
The left renal artery of the donor was cannulated for in situ perfusion (arrow). The isthmus was clamped during perfusion (arrowhead), and a divided fusion site of the isthmus was confirmed (dotted line).

The surgery was performed in March 2013. A donor exploration was performed through an abdominal midline incision. The accessory arteries were ligated. The left kidney was procured by clamping and ligation of the left renal artery. To demarcate the boundary, the left renal artery was cannulated for in situ perfusion with perfusion solution. Because the lower vein drained the left and right kidneys, the perfused boundary of the isthmus was unclear. Furthermore, the isthmus was clamped during perfusion, and the boundary of the isthmus was confirmed (Figure 3). The kidney was divided at the left margin of the perfused boundary, and the left renal vein was ligated. The cut ends were sealed with polyglactin sutures, and the parenchyma approximated. After unclamping the isthmus in the donor, no bleeding and no urine leakage were detected from transacted surfaces.

At the back table, the superior pole renal artery was anastomosed side-to-side with the inferior renal artery. To detect injury of the vessels, indigo carmine was administered through the renal artery. Moreover, to detect injury of the collecting system and prevent urine leakage, indigo carmine was administered through the ureter. Detected injuries of vessels and collecting systems were closed with poliglecaprone sutures. The cut ends were sealed with polyglactin sutures, and the parenchyma was approximated.

**Figure 4:**
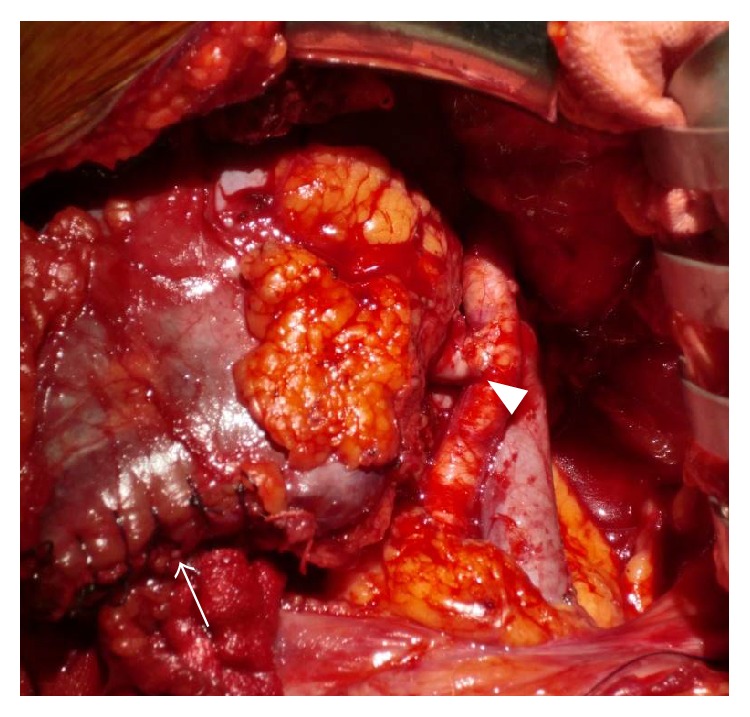
The horseshoe kidney after transplantation into the recipient. The edge of the isthmus was sutured (arrows). The renal artery was anastomosed to the external iliac artery (arrowhead), and the renal vein was anastomosed to the external iliac vein.

The transplant was performed in the right iliac fossa of the recipient. The renal artery was anastomosed to the external iliac artery, and the renal vein was anastomosed to the external iliac vein (Figure 4). The ureter was connected to the bladder using a Lich-Gregoir technique with a ureteral stent placed in situ. After perfusion of the graft, no bleeding and no urine leakage were detected from the transacted surfaces. After 191 min of renal ischemia, including 6 min of warm ischemia, the graft began producing urine. No complications were observed after surgery. Induction therapy consisted of 20 mg basiliximab administered on days 0 and 4, and cyclosporine, mycophenolate mofetil, and methylprednisolone were initiated for maintenance immunosuppression. The patient required control of hypertension by oral medication and hyperglycemia by insulin preparation after transplantation. Subsequently, he had no indications of surgical complications or rejection and was discharged 28 days after surgery with a serum creatinine level of 2.04 mg/dL. At 22 months' follow-up, the patient maintained serum creatinine levels between 2.00 and 2.20 mg/dL.

**Figure 5:**
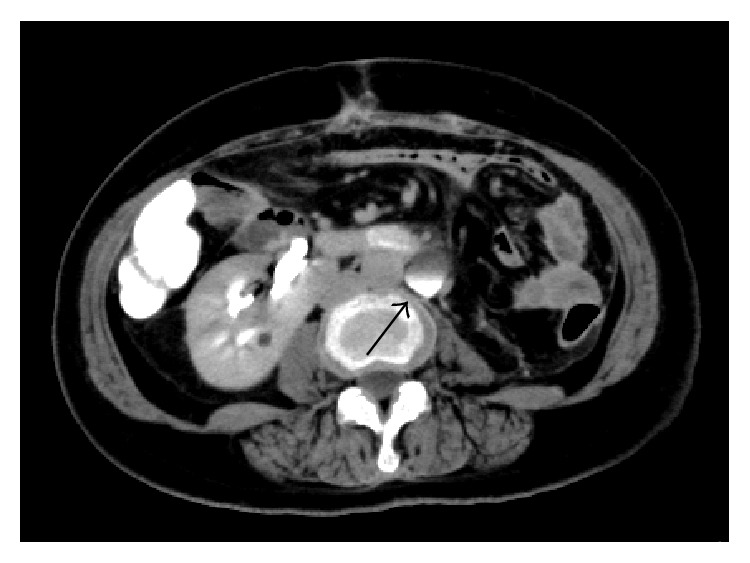
Postoperative donor CT images with contrast enhancement showed a site of fluid collection on the cut edge of the isthmus (arrow).

After donor nephrectomy, asymptomatic fluid collection, which was thought to be derived from the collecting system, occurred on the cut surface at the residual isthmus for a while (Figure 5) and decreased following a natural course 1 month after surgery. The donor's renal function has not worsened with serum creatinine levels between 0.53 and 0.66 mg/dL.

## 3. Discussion

A horseshoe kidney is the most common anatomic variation of the kidney, with an incidence of 1 in 600–800 adults [1]. Most horseshoe kidneys are fused at the lower poles, causing an incomplete rotation. Hence, the renal pelvises are situated in a ventral position, and the course of the ureter follows in front of the lower pole. In two-thirds of cases with horseshoe kidneys, complications occur based on ureteropelvic junction obstruction, such as hydronephrosis, renal calculi, and urinary tract infection. The vascular anatomy of horseshoe kidneys is usually complex, with only 30% of all horseshoe kidneys having a single renal artery to each side. The positions as well as the numbers of renal arteries and veins vary greatly, while the renal isthmus often has a separate blood supply. The renal isthmus connecting the pole of a horseshoe kidney is mostly composed of functional parenchyma.

Transplantation of a horseshoe kidney was first reported by Nelson and Palmer in 1975 [2]. Following their report, a few reviews of horseshoe kidney transplantation have been published [3, 4]. The horseshoe kidney from a deceased donor can be transplanted en bloc or split for 1 or 2 recipients after division. There is no consensus regarding the outcome associated with using a transplanted horseshoe kidney. Some authors claim that results from horseshoe kidney transplantation appear to be similar to those of normal kidney transplantation [3]. On the other hand, some authors have reported that horseshoe kidney transplantation is associated with a higher percentage of primary nonfunction [4], and the results of transplants using horseshoe kidneys are worse than those for normal kidneys [5].

**Figure 6:**
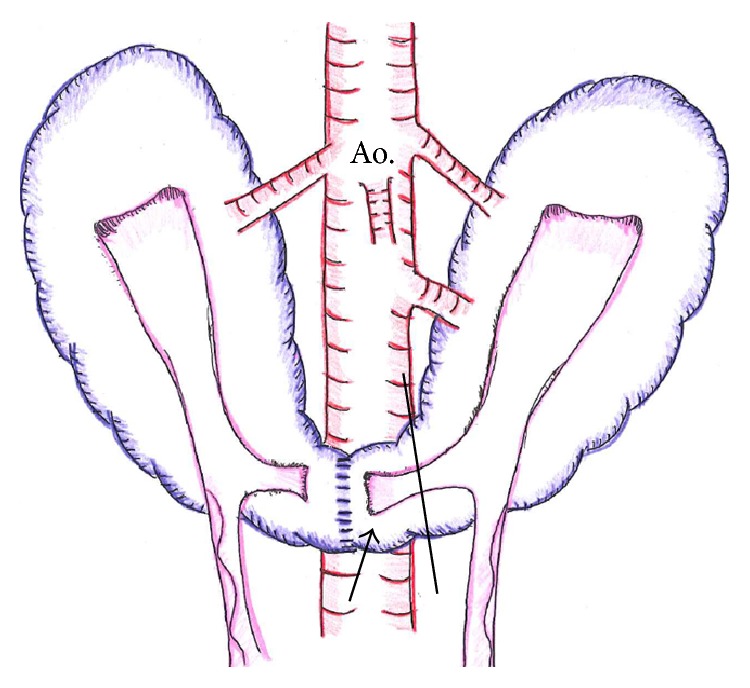
Illustration shows the horseshoe kidney with blood supplied from the aorta (Ao.) and collecting systems. The isthmus was divided on the left side position from the center (full line). A part of the left collecting system was preserved on the isthmus of the donor side (arrow).

A literature review revealed that six horseshoe kidneys were transplanted from living donors [5–9]. Among these, urine leakage occurred during the postoperative period in three cases. All transplanted kidney allografts demonstrated good function at a median follow-up of 14 months (range, 6–30 months). In living donors, the decision regarding where to divide the kidney is best made after the vascular anatomy and the anatomy of the collecting systems has been meticulously evaluated preoperatively using urography and CT. When the urinary collecting systems of the two sides are not connected and do not overextend into the contralateral part of the horseshoe kidney, the isthmus can be safely divided. If the urinary collecting system of a horseshoe kidney crosses the midline and the isthmus is divided, one or more calices are severed. This condition can be complicated by a urinary fistula, which is intractable. In the split technique, most surgeons transect the parenchyma according to the demarcation line made via methylene blue administration into the arterial system. Although the urinary collection system is frequently injured during separation of a renal isthmus, intraoperative perfusion of the graft with methylene blue into the renal artery was not sufficient to detect leakage of the urinary system in the literature. Therefore, we used a different technique for the split procedure. First, we ligated the inferior accessory artery and perfused the left renal artery in situ. After clamping the isthmus along the demarcation line, the parenchyma was transected (Figure 6). Furthermore, we administered indigo carmine through the renal artery and ureter to detect injury to the vessels and collecting system at the back table. In addition, to prevent complications such as urine leakage or hemorrhage after dividing the parenchyma in a living donor, the renal isthmus was divided at the left margin of the perfused boundary. In our case, there were no vascular complications due to multiple vascular abnormalities. Although slight urine leakage occurred from the cut end of the isthmus after donor nephrectomy, this fluid leakage was asymptomatic and decreased immediately.

Our case revealed that the horseshoe kidney can be an appropriate organ for transplantation. Because the number of patients awaiting a kidney transplant increases while the donor pool remains limited, we believe that using a horseshoe kidney as a renal allograft after a detailed preoperative evaluation may contribute to an expanded donor pool.
